# UK Multicenter Prospective Evaluation of the Leibovich Score in Localized Renal Cell Carcinoma: Performance has Altered Over Time

**DOI:** 10.1016/j.urology.2019.09.044

**Published:** 2020-02

**Authors:** Naveen S. Vasudev, Michelle Hutchinson, Sebastian Trainor, Roisean Ferguson, Selina Bhattarai, Adebanji Adeyoju, Jon Cartledge, Michael Kimuli, Shibendra Datta, Damian Hanbury, David Hrouda, Grenville Oades, Poulam Patel, Naeem Soomro, Grant D. Stewart, Mark Sullivan, Jeff Webster, Michael Messenger, Peter J. Selby, Rosamonde E. Banks

**Affiliations:** 1 Leeds Institute of Medical Research at St James's, St. James's University Hospital, Leeds, UK; 2 Department of Pathology, St James's University Hospital, Leeds, UK; 3 Stockport NHS Foundation Trust, Stockport, UK; 4 Department of Urology, St James's University Hospital, Leeds, UK; 5 University Hospital of Wales, Cardiff Heath Park, Cardiff, Wales; 6 Lister Hospital, Stevenage, UK; 7 Charing Cross Hospital, Imperial College Healthcare NHS Trust, London, UK; 8 Queen Elizabeth University Hospital, Glasgow, UK; 9 Divison of Cancer & Stem Cells, School of Medicine, University of Nottingham, Nottingham, UK; 10 Newcastle Upon Tyne Hospitals NHS Foundation Trust, Newcastle upon Tyne, UK; 11 NHS Lothian, Edinburgh, UK; 12 Oxford University Hospitals NHS Foundation Trust, Oxford, UK; 13 Northwick Park Hospital, Harrow, UK

## Abstract

**Objective:**

To examine changes in outcome by the Leibovich score using contemporary and historic cohorts of patients presenting with renal cell carcinoma (RCC)

**Patients and Methods:**

Prospective observational multicenter cohort study, recruiting patients with suspected newly diagnosed RCC. A historical cohort of patients was examined for comparison. Metastasis-free survival (MFS) formed the primary outcome measure. Model discrimination and calibration were evaluated using Cox proportional hazard regression and the Kaplan-Meier method. Overall performance of the Leibovich model was assessed by estimating explained variation.

**Results:**

Seven hundred and six patients were recruited between 2011 and 2014 and RCC confirmed in 608 (86%) patients. Application of the Leibovich score to patients with localized clear cell RCC in this contemporary cohort demonstrated good model discrimination (*c*-index = 0.77) but suboptimal calibration, with improved MFS for intermediate- and high-risk patients (5-year MFS 85% and 50%, respectively) compared to the original Leibovich cohort (74% and 31%) and a historic (1998-2006) UK cohort (76% and 37%). The proportion of variation in outcome explained by the model is low and has declined over time (28% historic vs 22% contemporary UK cohort).

**Conclusion:**

Prognostic models are widely employed in patients with localized RCC to guide surveillance intensity and clinical trial selection. However, the majority of the variation in outcome remains unexplained by the Leibovich model and, over time, MFS rates among intermediate- and high-risk classified patients have altered. These findings are likely to have implications for all such models used in this setting.

Numerous changes have taken place over the past 2 decades in relation to the diagnosis and management of patients presenting with renal cell carcinoma (RCC). Alongside significant continued projected increases in incidence,^1^ rates of obesity and tobacco smoking, established RCC risk factors, have altered^2^, ^3^, ^4^ and there has been a general shift away from radical nephrectomy (RN) to partial nephrectomy (PN),^5^ and minimally invasive instead of open procedures.^6^

Outcomes in patients postnephrectomy for localized RCC are highly variable, but risk stratification tools have not evolved during this same period, remain reliant on clinicopathologic criteria alone and typically explain only a small proportion of the observed variance in outcomes.^7^ As clinical practice and tumor biology changes, so too can the performance of such models and agreement between observed and predicted outcomes may shift over time. Indeed, recently reported trials of adjuvant therapy, conducted across North America and Asia, in patients deemed at high risk of relapse based on tumor stage and grade, have shown better than expected disease-free survival rates among placebo-treated patients, when compared to historical data.^8^, ^9^, ^10^

In Europe, the most widely used risk stratification tool is the Leibovich score, developed in 2003 in patients undergoing RN between 1970 and 2000 at a single high-volume US centre.^11^ Its performance in a contemporary, prospective, multi-institutional European cohort has not been assessed. We conducted a National Institute for Health Research funded prospective observational multicenter cohort study to generate a high-quality biobank with associated clinical data and follow-up for the evaluation of novel and emerging prognostic RCC biomarkers.^12^ Here, utilizing both this cohort and a distinct historical group of UK patients, we examine outcomes by the Leibovich score and reveal alteration in performance of the model over time.

## METHODS

### Patients and Samples

Patients from 11 UK centers with newly diagnosed suspected RCC, of all stages and histologic types, with no prior treatment, were eligible. Exclusion criteria were those with known familial RCC (eg, VHL syndrome), renal cancer acquired following and/or during renal replacement therapy and those at high risk or with known HIV, Hepatitis B/C or other blood-borne infectious disease. Patients undergoing any procedure, including ablation, radical or PN or biopsy only, were eligible for the overall study, although only nephrectomized patients were included in the current analysis. Baseline clinical, biochemical, and hematological data were collected, together with follow-up data, all using standardized case report forms and co-ordinated through the Leeds Clinical Trials Research Unit. As part of the study, and following informed written consent, baseline blood and urine samples and an Formalin fixed paraffin embedded (FFPE) tumor tissue block (from patients undergoing nephrectomy) were also collected. The study was approved by the Local Research Ethics Committee (ethical approval 10/H1306/6). A historical cohort, composed of patients attending St James's University Hospital, Leeds between 1998 and 2006, who had been prospectively recruited to a local biobanking study using the same eligibility and exclusion criteria as above were also included as a separate cohort for comparison.

### Pathology

Original pathology reports were requested and tumor type, stage, size, and lymph node status, as well as presence or absence of necrosis and sarcomatoid and/or rhabdoid change, extracted. For clear cell RCC (ccRCC) cases only, the Leibovich score was also calculated.^11^ Baseline imaging (CT/MRI) reports were also reviewed.

### Statistical Methods

Metastasis-free survival (MFS) was calculated for patients with localized disease, defined as the period from date of nephrectomy to date of distant recurrence. Patients without recurrence were censored at the date they were last known to be recurrence free (for patients who died without recurrence this was date of death).

Patient characteristics in the contemporary and historic cohorts were compared using Wilcoxon rank-sum and chi-squared tests. Where information was available, categorical variables were compared with data from the original Leibovich cohort.

Performance of the Cox proportional hazard (PH) model on which the Leibovich score is based was assessed in terms of discrimination, calibration,^13^ and estimation of explained variation (EV).^14^ Cox PH models with Leibovich risk group as predictor were used to estimate hazard ratios and *c*-index to assess discrimination. The Kaplan-Meier method was used to estimate and visualize MFS to assess calibration. EV was calculated as described^14^ using downloadable R scripts^15^ adapted in house. In addition to an estimate of EV for the model as a whole, this method allows for the approximation of the proportion of EV which can be attributed to individual model elements in both the univariate (unadjusted EV) and multivariable (adjusted EV) setting. Unadjusted EV was calculated by including each of the Leibovich score elements, in turn, into a univariate Cox PH model with MFS as the response variable. Adjusted EV was calculated as the difference in EV between the Cox PH model including all elements of the Leibovich score, and the multivariable model excluding each of the elements in turn, to give an estimate for each variable when adjusting for the others.

All statistical tests were 2-sided, all analyses were undertaken in the R environment for statistical computing.^16^

## RESULTS

In total, 706 patients were recruited to the study between July 2011 and June 2014 from 11 UK centers. An RCC was subsequently confirmed in 608 (86%) cases with 79% of these being clear cell, 10% papillary, 8% chromophobe, and 3% unclassified tumors. Characteristics for all RCC patients are shown in Supplementary Table S1 with details of patients found not to have RCC shown in Supplementary Table S2. A flow diagram of patients is presented in Supplementary Figure S1.

### RCC Patients

#### Leibovich Score Performance

The performance of the Leibovich score was assessed using the same inclusion and exclusion criteria as the original report,^11^ with the exception that patients undergoing PN were included in our analyses to reflect current practice. Of the 480 ccRCC patients in our contemporary UK cohort, 384 were eligible and had complete data to allow calculation of the Leibovich score and MFS. For comparison, and to examine changes over time in UK cohorts, we examined the performance of the Leibovich score in a historical cohort of 191 patients undergoing nephrectomy for ccRCC between 1998 and 2006 at a single UK institution (Leeds).

Clinical and demographic characteristics of the contemporary ccRCC subcohort and historical cohort are shown in Table 1, alongside available patient characteristics from the original Leibovich cohort. Median follow-up in the contemporary and historic UK cohorts was 4.4 years (IQR: 3.4, 5.2) and 10.7 years (IQR: 7.87, 12.71), respectively. Certain elements of the model, such as tumor necrosis and lymph node status, have remained constant over time. However, shifts in the distribution of tumor grade, size, and pT stage are evident, which start to become apparent when comparing the Leibovich cohort (1970-2000) with the historic UK cohort (1998-2006) and persist in the contemporary UK cohort (2011-2014). These differences would, however, be expected to be accounted for by the model.

**Table 1:** Characteristics of nephrectomized localized ccRCC patients included in analysis

		Cohorts		
Characteristic		Contemporary UK 2011-2014 *n* = 384	Historic UK 1998-2006 *n* = 191	Original Leibovich 1970-2000 *n* = 1671
Age at procedure	Median (range)	63 (29, 92)	64 (29, 86)	65 (24, 89)
Gender	Male	252 (66)	110 (58)	1061 (64)
	Female	132 (34)	81 (42)	610 (36)
Procedure†^,^‡^,^§	PN	100 (26)	12 (6)	0 (0)
	RN	284 (74)	178 (93)	1671 (100)
	Missing	0 (0)	1 (1)	0 (0)
Stage‡	I	227 (59)	93 (49)	NA
	II	42 (11)	13 (7)	NA
	III	115 (30)	85 (45)	NA
Leibovich Score elements				
Tumor size (mm)	Median (range)	50 (11, 180)	55 (2, 160)	65 (8, 240)
Tumor size†^,^§	≤10 cm	350 (91)	166 (87)	1312 (78)
	>10 cm	34 (9)	25 (13)	359 (22)
Grade†^,^§	1	6 (2)	6 (3)	182 (11)
	2	131 (34)	61 (32)	786 (47)
	3	200 (52)	94 (49)	600 (36)
	4	47 (12)	30 (16)	103 (6)
pT*^,^†^,^‡^,^§	1a	126 (33)	45 (24)	384 (23)
	1b	102 (27)	48 (25)	440 (26)
	2	42 (11)	16 (8)	335 (20)
	3	114 (29)	82 (43)	507 (30)
	4	0 (0)	0 (0)	5 (1)
pN	0/X	376 (98)	181 (95)	1605 (96)
	1	8 (2)	10 (5)	56 (3)
	2	0 (0)	0 (0)	10 (1)
Necrosis	No	282 (73)	146 (76)	1232 (74)
	Yes	102 (27)	45 (24)	439 (26)
*Leibovich risk group*				
	Low	150 (39)	60 (31)	689 (41)
	Intermediate	163 (42)	86 (45)	608 (36)
	High	71 (19)	45 (24)	374 (22)

NA, not available; PN, partial nephrectomy; RN, radical nephrectomy.

⁎ AJCC 2002 TNM staging applied in original Leibovich and historic UK cohorts vs 2010 TNM staging applied to contemporary UK cohort.

† *P* <.05 Leibovich vs contemporary UK cohort.

‡ *P* <.05 contemporary UK vs historic UK cohort.

§ *P* <.05 Leibovich vs historic UK cohort.

The *c*-index of the Cox PH model with risk group as the predictor in the contemporary UK cohort was 0.77 (SE: 0.04) and in the historic UK cohort was 0.73 (SE: 0.04), demonstrating good model discrimination, further evidenced by corresponding hazard ratios in the intermediate- and high-risk groups; 5.11, 95% CI: (1.77-14.8), and 23.4, 95% CI: (8.30-66.0), respectively, in the contemporary cohort and 4.22, 95% CI: (1.62-11.0) and 16.1, 95% CI: (6.16-42.2), in the historic cohort (with low risk the reference).

**Figure 1:**
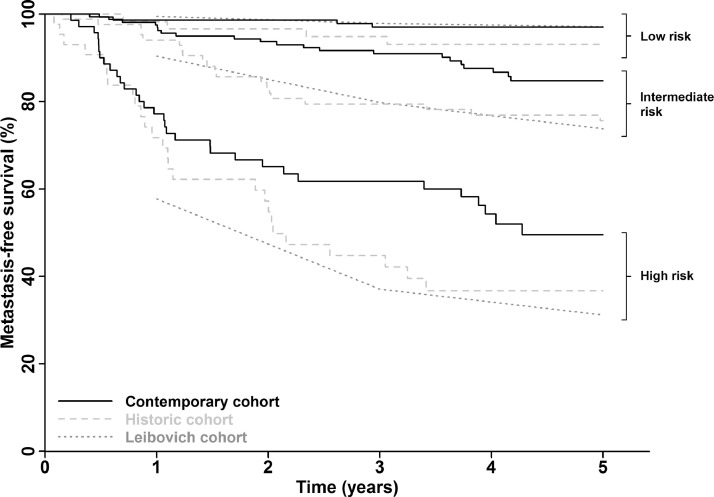
Metastasis-free survival by Leibovich risk group. Survival curves are shown for patients in the contemporary UK cohort, a comparative historical UK cohort and the original US cohort as reported by Leibovich et al.^11^

Figure 1 shows Kaplan-Meier curves for the risk groups in both UK cohorts and approximate survival curves estimated from MFS figures reported in the original Leibovich paper, and comparative 1-, 3-, and 5-year MFS rates are shown in Table 2. The Kaplan-Meier plots confirm the discriminative ability of the model to differentiate between risk groups. The agreement of the survival curves between the historic and original Leibovich cohorts suggest a degree of calibration, however, the survival curves for the contemporary cohort in the intermediate- and high-risk groups are not well aligned with the Leibovich data curves, indicating some degree of miscalibration. This is also evident in the observed MFS rates. For example, at year 5, while MFS rates in the high-risk group in the original and historic cohorts are similar (31% and 37%, respectively), it is higher in our contemporary high-risk group (50%).

**Table 2:** 1-, 3-, and 5-year metastasis-free survival rates by Leibovich risk group

	Cohorts		
Leibovich Risk Group	Contemporary UK *n* = 384	Historic UK *n* = 191	Original Leibovich *n* = 1671
*1-year MFS rate (%) SE*			
Low	99 ± 1	98 ± 2	99.5 ± 0.3
Intermediate	98 ± 1	94 ± 3	90.4 ± 1.2
High	77 ± 5	72 ± 7	57.7 ± 2.6
*3-year MFS rate (%) SE*			
Low	97 ± 1	95 ± 3	97.9 ± 0.6
Intermediate	91 ± 2	79 ± 4	79.8 ± 1.7
High	62 ± 6	45 ± 8	37.1 ± 2.7
*5-year MFS rate (%) SE*			
Low	97 ± 1	93 ± 3	97.1 ± 0.7
Intermediate	85 ± 3	76 ± 5	73.8 ± 2.0
High	50 ± 7	37 ± 8	31.2 ± 2.7

SE, standard error.

The percentage of variation explained by the Cox PH model on which the Leibovich score is based was 28% in the historic UK cohort and 22% in the contemporary UK cohort (Table 3). The majority of EV (unadjusted and adjusted) was attributable to tumor stage in both cohorts, while the contribution of tumor grade and presence of necrosis to the EV was observed to decrease with time.

**Table 3:** Proportion of explained variation by UK cohort. Estimate of explained variation (EV) for the full model and that which can be attributed to individual model elements in both the univariate (unadjusted EV) and multivariable (adjusted EV) setting are presented

Historic UK cohort		Unadjusted explained variation (%)	Adjusted explained variation (%)
	pT	16.84	5.35
	pN	2.47	0.20
	Tumor ≥100 mm	5.91	0.47
	Grade	12.53	2.97
	Necrosis	14.07	3.75
	Full model	27.95	
Contemporary UK cohort		Unadjusted explained variation (%)	Adjusted explained variation (%)
	pT	14.86	6.95
	pN	3.33	0.13
	Tumor ≥100 mm	5.88	0.57
	Grade	8.01	1.48
	Necrosis	8.53	1.11
	Full model	21.75	

## DISCUSSION

Our ability to deliver effective patient centered cancer care depends substantially on our ability to estimate likely patient outcomes to aid our planning and as part of shared decision taking. Although clinicopathologic models have become widely incorporated into clinical pathways to guide decision making, it should be recognized that the performance of such models may alter over time and must, therefore, be periodically re-examined. Here, by example, we show that the performance of the Leibovich score has altered with time, carrying potential implications for other prognostic models, developed more than a decade ago, both in localized RCC,^17^, ^18^, ^19^ and other settings.

The discriminative ability of the Leibovich model appears to have been retained over time, with a similar *c*-index in both the current cohort as well as in a historical UK cohort, which we examined for comparative purposes and to try to control for factors other than time of recruitment (such as a UK vs US setting). Furthermore, the distribution of patients across low-, intermediate-, and high-risk groups has remained constant. Although absence of baseline survival function (or raw data) from the original Leibovich study limits options with regards to assessing model calibration, our indirect assessment of calibration implies a decline over time. While MFS rates were remarkably similar between the original Leibovich cohort and our historical cohort, patients in our contemporary cohort demonstrated reduced relapse rates among intermediate- and high-risk groups. For example, the 5-year MFS among high-risk patients in the original Leibovich, historic and contemporary cohorts was 31.2%, 37%, and 50%, respectively.

This is important given that the Leibovich score is widely employed in the clinic to counsel patients, guide intensity of follow-up and for the design and powering of adjuvant studies. Ongoing phase III trials (eg, NCT03288532), examining the efficacy of adjuvant checkpoint inhibitors, include patients with intermediate-risk disease as defined by the Leibovich score, although our findings suggest that the majority (85%) of these patients remain metastasis free at 5 years, and therefore likely cured, through surgery alone. Given the associated costs, resource implications and potential toxicity of immunotherapy, it is imperative that patient selection is optimized and that the performance of risk stratification tools in the population in which they are being applied is understood and accounted for.

The reasons for the improvement in MFS rates and alteration in performance of the model over time are uncertain. It is likely, however, that changes in practice, such as advances in imaging, improvements in surgical techniques and refinement of grading and classification of tumors that have taken place over the past 2 decades are, at least in part, responsible. Retrospective vs prospective data collection, varying geographical location and methodological differences may also be considered, although we have tried to account for this by including a historical UK cohort of patients and by replicating the original study design as closely as possible. It is also important to recognize that, while the elements making up the Leibovich score represent independent prognostic factors in patients with resected ccRCC,^20^ the importance of even such strong prognostic factors in determining outcome at an individual patient level is often low.^7^ Methods to quantify this (termed explained variance) have long been developed and recently highlighted,^7^ although as a concept remains poorly understood and underutilized. We found that the Leibovich model accounted for just 28% of the observed variance in MFS in our historical cohort, declining to 22% among contemporary patients. Thus, the majority of the variance in outcomes remains unexplained by the model and refinement limited to consideration of additional clinical factors alone^21^ seems unlikely to meaningfully improve this situation. Differences in molecular tumor biology, for example, that are likely to be critical in determining individual outcomes, remain unaccounted for and poorly defined. Even small changes in these unknown variables over time, conceivably in this case due to shifts in rates of obesity, smoking, and hypertension, are likely to significantly impact the performance of a given model.

Only a small number of studies have examined the performance of the Leibovich score since its original description. These include a retrospective study among Asian patients (*n* = 355) undergoing nephrectomy between 1990 and 2006^22^ and a second, much larger, retrospective European single institution study of patients undergoing nephrectomy between 1984 and 2006.^23^ The discriminative ability of the Leibovich score was confirmed in both studies, with 5-year DFS rates of 76.8% and 33.6% for intermediate- and high-risk groups in the former study, which are comparable to those originally reported.^11^^,^^22^ A more contemporary, but again retrospective, study of 386 patients conducted in Norway between 1993 and 2013 reported suboptimal calibration for patients in the intermediate- and high-risk groups, with a 5-year relapse-free survival among high-risk patients of 41.2%, in support of our current findings.^24^ More recently still, the performance of 8 different prognostic models, including the Leibovich score, has been reported among US patients recruited to the phase III adjuvant ASSURE trial between 2006 and 2010.^25^ The 5-year MFS rates were 79.6% (95% CI: 76.5-82.4) and 61.8% (57.2-66.1) among Leibovich-score defined intermediate- and high-risk patients, respectively, with a *c*-index for the model of 0.625 (0.623-0.626).^26^ While our data demonstrate better maintained discriminatory ability of the Leibovich model, the observed improvement in MFS rates over time are consistent with our findings in our UK cohort of patients. Furthermore, the fact that the majority (95%) of patients in ASSURE underwent a RN suggests that the inclusion of patients undergoing a PN does not account for these differences.

The strengths of this study include its multicenter prospective design, with comprehensive baseline and follow-up data collection and the inclusion of a comparative historical cohort of UK patients. As one of the main study objectives, a translational biobank has been generated, to support the validation of prognostic and diagnostic RCC biomarkers. Limitations include the lack of central pathology review and a shorter median length of follow-up of 4.4 years in the current cohort, compared to 5.4 years as originally reported by Leibovich et al.^11^

## CONCLUSION

In summary, we provide the most contemporary assessment of the Leibovich score to date and show that outcomes for patients classified as being at intermediate- or high risk have altered over time. Our findings carry implications for this model, and potentially other nomograms applied in this or other settings, to guide clinical decision-making. Identification of patients destined to relapse remains suboptimal, highlighting the need for improved risk stratification tools.

## Appendix Supplementary materials

Supplementary Figure 1. Flow diagram of patients recruited to contemporary cohort. Image, image 1

Image, application 2

Image, application 3

## Footnotes

Supplementary material associated with this article can be found in the online version at https://doi.org/10.1016/j.urology.2019.09.044.
