# Supplementary figures and images for: UK Multicenter Prospective Evaluation of the Leibovich Score in Localized Renal Cell Carcinoma: Performance has Altered Over Time
Source: Urology. 2020 Feb;136:162–8. doi: 10.1016/j.urology.2019.09.044 (PMC7043004; doi:10.1016/j.urology.2019.09.044)

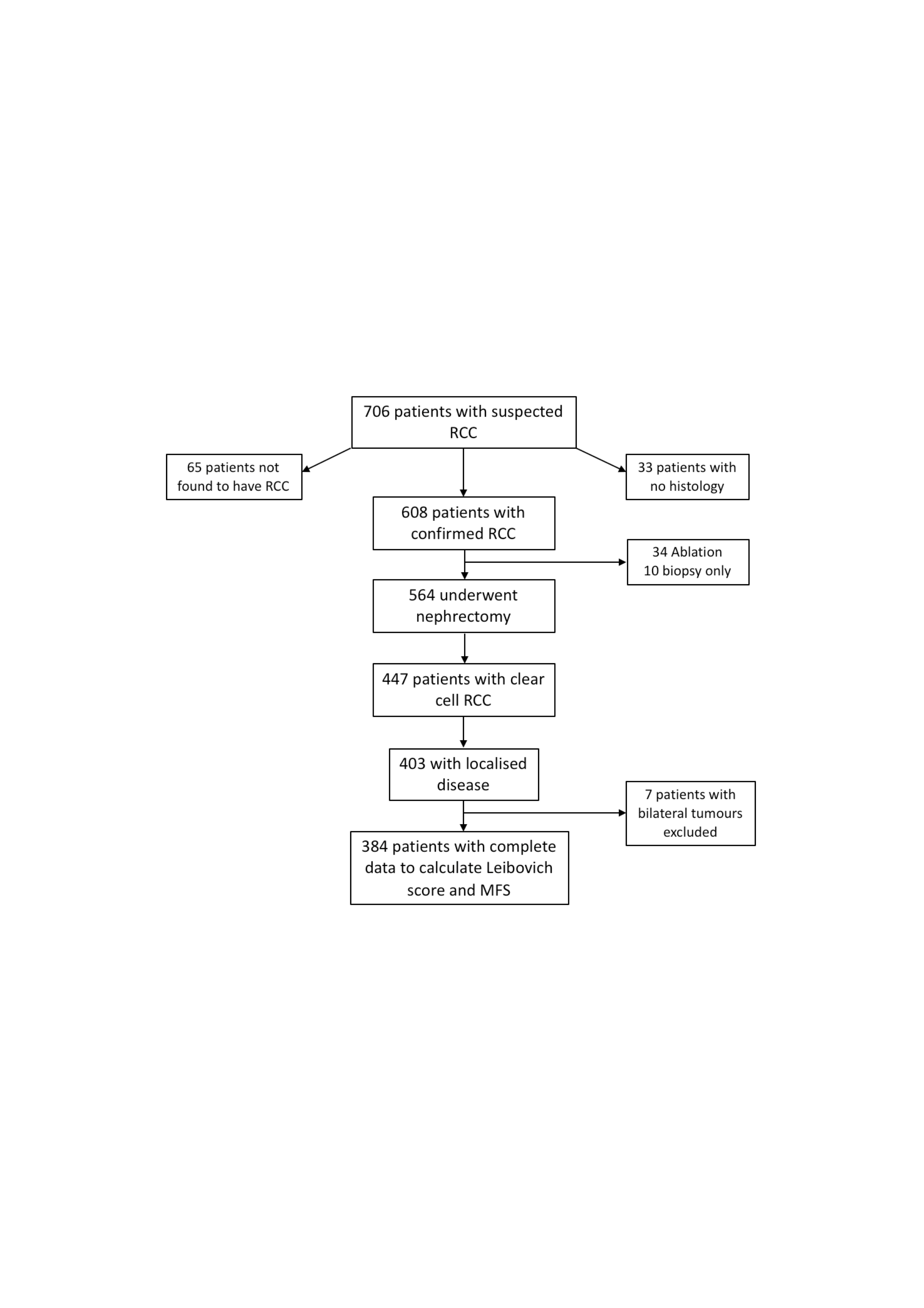

Supplement: Supplementary file 1 — Supplementary Figure 1. Flow diagram of patients recruited to contemporary cohort. [file mmc1.jpg]
