# Supplementary material for: UK Multicenter Prospective Evaluation of the Leibovich Score in Localized Renal Cell Carcinoma: Performance has Altered Over Time
Source: Urology. 2020 Feb;136:162–8. doi: 10.1016/j.urology.2019.09.044 (PMC7043004; doi:10.1016/j.urology.2019.09.044)
Supplement: Supplementary file 2 [file mmc2.docx]

**Supplementary Table 1. RCC Patient Characteristics – Contemporary cohort**

| Characteristic | | All n=608 (100%) |
| --- | --- | --- |
| Age at op (CT if no op) | median (range) | 63.50 (29.00, 92.00) |
| Gender | Male | 396 (65) |
|  | Female | 212 (35) |
| Ethnicity | White | 572 (94) |
|  | Other | 35 (6) |
|  | Missing | 1 (0) |
| BMI | median (range) | 28.10 (15.60, 74.40) |
| BMI categorised | BMI<24.9 | 153 (25) |
|  | 25<BMI<29.9 | 220 (36) |
|  | BMI≥30 | 230 (38) |
|  | Missing | 5 (1) |
| Smoking status | Current | 101 (17) |
|  | Past | 269 (44) |
|  | Passive | 14 (2) |
|  | Never | 222 (37) |
|  | Missing | 2 (0) |
| Alcohol consumption^a^ | Teetotal | 167 (27) |
|  | Light | 301 (50) |
|  | Moderate | 76 (12) |
|  | High | 59 (10) |
|  | Missing | 5 (1) |
| ECOG PS | 0 | 484 (80) |
|  | 1 | 103 (17) |
|  | 2 | 17 (3) |
|  | 3 | 2 (0) |
|  | 4 | 1 (0) |
|  | Missing | 1 (0) |
| Time from urology consultation to procedure | median (range) | 38.00 (0.00, 2148.00) |
| Procedure categorised | Nephrectomy | 564 (93) |
|  | Ablation | 34 (6) |
|  | Biopsy only | 10 (2) |
| Nephrectomy type | Laparoscopic | 404 (72) |
|  | Open | 152 (27) |
|  | Missing | 8 (1) |
|  | Not applicable (ablation or biopsy only) | 44 |
| Tumour type | Clear Cell | 480 (79) |
|  | Papillary | 60 (10) |
|  | Chromophobe | 46 (8) |
|  | Unclassified | 18 (3) |
|  | Translocation Carcinoma | 2 (0) |
|  | Chromophobe & Clear Cell (cystic) | 1 (0) |
|  | Mucinous Tubular and Spindle Cell | 1 (0) |
| Tumour size (mm) | median (range) | 55.00 (11.00, 240.00) |
| pT categorised^b^ | 1 | 341 (57) |
|  | 2 | 78 (13) |
|  | 3 | 172 (29) |
|  | 4 | 4 (1) |
|  | X | 2 (0) |
|  | Missing | 1 (0) |
|  | Not applicable (biopsy only) | 10 |
| pN | 0 | 66 (11) |
|  | 1 | 14 (2) |
|  | X | 528 (87) |
| pM | 0 | 597 (98) |
|  | 1 | 11 (2) |
| Grade | 1 | 10 (2) |
|  | 2 | 162 (28) |
|  | 3 | 282 (50) |
|  | 4**^c^** | 91 (16) |
|  | Missing | 24 (4) |
|  | Not applicable (ablation or biopsy only) | 39 |
| Necrosis | No | 364 (64) |
|  | Yes**^d^** | 201 (36) |
|  | Not applicable (ablation or biopsy only) | 43 |
| Microvascular invasion | No | 469 (83) |
|  | Yes | 95 (17) |
|  | Not applicable (ablation or biopsy only) | 44 |
| Sarcomatoid features | No | 528 (94) |
|  | Yes | 36 (6) |
|  | Not applicable (ablation or biopsy only) | 44 |
| Rhabdoid features | No | 533 (94) |
|  | Yes | 31 (6) |
|  | Not applicable (ablation or biopsy only) | 44 |
| Stage | I | 341 (56) |
|  | II | 70 (12) |
|  | III | 142 (23) |
|  | IV | 55 (9) |
| Leibovich Score categorised | Low risk | 154 (38) |
|  | Intermediate risk | 171 (42) |
|  | High risk | 80 (20) |
|  | Not applicable | 203 |

**^a^** Defined as: Teetotal: 0 units; Light: males <3 units/day and females < 2 units/day; Moderate: males 3-6 units/day and females 2-4 units/day; High: males >6 units/day and females >4 units/day. A unit of alcohol is defined as 10 g of alcohol

**^b^** All ablation patients were classed as pT1, based on CT tumour size

**^C^** Includes 5 patients with biopsy only, containing G4 tumour

**^d^** Includes one biopsy case
