# Supplementary material for: UK Multicenter Prospective Evaluation of the Leibovich Score in Localized Renal Cell Carcinoma: Performance has Altered Over Time
Source: Urology. 2020 Feb;136:162–8. doi: 10.1016/j.urology.2019.09.044 (PMC7043004; doi:10.1016/j.urology.2019.09.044)
Supplement: Supplementary file 3 [file mmc3.docx]

**Supplementary Table 2. Non-RCC lesions.** Nature of lesions in patients found not to have an RCC following recruitment to the study

|  | **Total (n=65)** |
| --- | --- |
| **Description** | **n (%)** |
| Abscess | 1 (1.5) |
| Adrenal Carcinoma | 1 (1.5) |
| Angiomyolipoma | 8 (13) |
| Cyst | 4 (6) |
| Cystic nephroma | 4 (6) |
| Hemangioblastoma | 1 (1.5) |
| Hematoma | 1 (1.5) |
| Inflammation and fibrosis only | 1 (1.5) |
| Leiomyomata | 1 (1.5) |
| Metanephric Adenoma | 2 (3) |
| Mixed Epithelioid Stromal tumor | 2 (3) |
| Myxoid mesenchymal tumor | 1 (1.5) |
| Necrosis | 1 (1.5) |
| Not cancer (NOS) | 1 (1.5) |
| Oncocytoma | 27 (42) |
| Pyelonephritis | 1 (1.5) |
| Rosai Dorfman disease | 1 (1.5) |
| Retroperitoneal smooth muscle cancer | 1 (1.5) |
| Solitary fibrous tumor | 1 (1.5) |
| Transitional cell carcinoma | 2 (3) |
| Wilm’s tumor | 2 (3) |
| Xanthogranulomatous pyelonephritis | 1 (1.5) |

A further 29/706 (4%) patients did not undergo a biopsy or nephrectomy and 4 (0.5%) patients were biopsied but no tumour was present in the cores
